# Autoimmune antibody decline in Parkinson’s disease and Multiple System Atrophy; a step towards immunotherapeutic strategies

**DOI:** 10.1186/s13024-017-0187-7

**Published:** 2017-06-07

**Authors:** Tomasz Brudek, Kristian Winge, Jonas Folke, Søren Christensen, Karina Fog, Bente Pakkenberg, Lars Østergaard Pedersen

**Affiliations:** 1 Research Laboratory for Stereology and Neuroscience, Bispebjerg-Frederiksberg Hospital, Copenhagen University Hospital, Bispebjerg, Bispebjerg Bakke 23, DK-2400 Copenhagen N, Denmark; 2 Department of Neurology, Bispebjerg-Frederiksberg Hospital, Copenhagen University Hospital, Bispebjerg, Bispebjerg Bakke 23, DK-2400 Copenhagen N, Denmark; 3 Bispebjerg Movement Disorders Biobank, Bispebjerg-Frederiksberg Hospital, Copenhagen University Hospital, Bispebjerg, Bispebjerg Bakke 23, DK-2400 Copenhagen N, Denmark; 4 Institute of Clinical Medicine, Faculty of Health, University of Copenhagen, Copenhagen, Denmark; 5 H. Lundbeck A/S, Ottiliavej 9, DK-2500 Valby Copenhagen, Denmark

**Keywords:** Parkinson’s disease, Multiple System Atrophy, Alpha-synuclein, Synuclein, Autoantibodies, Plasma

## Abstract

**Background:**

Parkinson’s’ disease (PD) and Multiple System Atrophy (MSA) are progressive brain disorders characterized by intracellular accumulations of α-synuclein and nerve cell loss in specific brain areas. This loss causes problems with movement, balance and/or autonomic functions. Naturally occurring autoantibodies (NAbs) play potentially an important role in clearing or/and blocking circulating pathological proteins. Little is known about the functional properties of anti-α-synuclein NAbs in PD and MSA, and there have been opposing reports regarding their plasma concentrations in these disorders.

**Methods:**

We have investigated the apparent affinity of anti-α-synuclein NAbs in plasma samples from 46 PD patients, 18 MSA patients and 41 controls using competitive enzyme-linked immunosorbent assay (ELISA) and Meso Scale Discovery (MSD) set-ups.

**Results:**

We found that the occurrence of high affinity anti-α-synuclein NAbs in plasma from PD patients is reduced compared to healthy controls, and nearly absent in plasma from MSA patients. Also, levels of α-synuclein/NAbs immunocomplexes is substantially reduced in plasma from both patient groups. Further, cross binding of anti-α-synuclein NAbs with β- and γ-synuclein monomers suggest, the high affinity anti-α-synuclein plasma component, seen in healthy individuals, is directed mainly against C-terminal epitopes. Furthermore, we also observed reduced occurrence of high affinity anti-phosphorylated-α-synuclein NAbs in plasma from PD and MSA patients.

**Conclusions:**

One interpretation implies that these patients may have impaired ability to clear and/or block the effects of pathological α-synuclein due to insufficient/absent concentration of NAbs and as such provides a rationale for testing immune-based therapeutic strategies directed against pathological α-synuclein. Following this interpretation, we can hypothesize that high affinity autoantibodies efficiently bind and clear potentially pathological species of α-synuclein in healthy brain, and that this mechanism is impaired or absent in PD and MSA patients.

## Background

Abnormal intracellular aggregation of α-synuclein is a pathological hallmark of Parkinson’s disease (PD), where α-synuclein is a major constituent of neuronal Lewy bodies, and Multiple System Atrophy (MSA), where α-synuclein aggregates are found mainly in oligodendroglia [1–3]. Both PD and MSA are associated with the degeneration of nerve cells in specific areas of the brain [4–6]. PD is the second most common neurodegenerative disorder among the elderly; only symptomatic therapeutic interventions based on dopamine replacement are available for the treatment of PD patients [7]. Whereas, MSA is relatively rare, it progresses more rapidly than does PD and treatment is less effective on motor symptoms, leading to death in less than ten years after diagnosis [8, 9]. Although α-synuclein is frequently assumed to be etiologically involved in these conditions, the mechanisms driving α-synuclein aggregation and their relationship to disease progression and neuronal degeneration are poorly understood [10].

A growing body of evidence from clinical studies and preclinical animal models emphasize a role of the immune system in the pathophysiology of PD [11, 12]. Naturally occurring autoantibodies (NAbs) make up two-thirds of the human antibody pool, and may be involved in diverse aspects of immunological reactions including regulatory and protective functions [13–15]. NAbs are now understood to have access to the central nervous system, where they are implicated in maintaining homeostasis by removing cell debris and in preventing inflammation by binding and neutralizing cytokines [16, 17]. A neuroprotective action of anti-α-synuclein antibodies has been demonstrated in several animal models of PD. For example, transgenic mice that produced high affinity antibodies following immunization with human α-synuclein displayed reduced intraneuronal accumulation of aggregated human α-synuclein, and decreased degeneration of dopamine neurons, apparently through promoting degradation and clearance of aggregated human α-synuclein via lysosomal pathways [18]. Also, antibodies specific for misfolded α-synuclein, have been found to block uptake and propagation of α-synuclein pathology in cell culture and in a mouse model [19]. Another study showed that active immunization using α-synuclein peptides reduced the accumulation of α-synuclein oligomers (but not monomers) and ameliorated the behavioral and neurodegenerative pathology in two different transgenic models of synucleinopathies [20, 21]. Furthermore, passive immunization studies using high affinity α-synuclein antibodies have been successful in ameliorating α-synuclein pathology and/or behavior in animal models [as reviewed by Bergstrom et al. [22]].

The titre of anti-α-synuclein NAbs in serum or plasma from PD patients has been the focus of previous investigations. Whereas some studies showed non-significant differences between PD patients and controls groups [23–25], others have shown increased [26–28] or decreased [29] α-synuclein NAbs levels in PD patients. To our knowledge none of these reports investigated the functional characteristics of anti-α-synuclein NAbs from PD patients, nor have there been any reports on anti-α-synuclein NAbs in MSA.

In general, the biological activity of an antiserum is influenced not only by its concentration of specific antibodies but also by its functional characteristics such as affinity and/or avidity. Thus, the analysis of NAbs in plasma antibody titer as well as affinity/avidity of the antibodies is required. Yanamandra et al. has reported that the immunoreactivity to α-synuclein is decreased in late PD, which manifests in fewer patients exhibiting marked immune responses towards α-synuclein [28].

We now report that the frequency of a high apparent affinity phenotype of anti-α-synuclein NAbs in plasma of PD patients is significantly reduced compared to healthy individuals, whereas these antibodies are nearly undetectable in plasma of MSA patients. In contrast, all tested individuals had abundance of anti-α-synuclein NAbs of low apparent affinity reflecting unspecific binding (affinity range > 1000 nM) or low affine polyreactivity (100-1000 nM). We hypothesize that high affinity auto-antibodies of the IgG sub-type efficiently bind and clear potentially pathological species of α-synuclein in healthy brain, and that this mechanism is impaired or absent in PD and MSA patients.

## Methods

### Patients and healthy control individuals

All PD and MSA patients were followed by a movement disorder specialist at the Movement Disorders Clinic, Department of Neurology, Bispebjerg-Frederiksberg Hospital, Copenhagen. The clinical diagnosis for PD was defined according to UK Parkinson’s Disease Society Brain Bank clinical diagnostic criteria [30]. The clinical diagnosis of MSA was performed according to the accepted clinical diagnostic criteria [31] and with initial inclusion of cases with both possible and probable diagnoses (either the parkinsonian or the cerebellar subtype). Patients were included consecutively and a clinical follow-up was performed for all patients. At follow-up only those patients fulfilling the diagnostic criteria for a probable or definite MSA (*n* = 18) or definite PD (*n* = 46) diagnosis were accepted and included in the study. All PD and MSA patients received levodopa treatment. Healthy control volunteers (*n* = 41) were recruited through general announcements or advertisements at the hospital and were free of conditions that might affect the central nervous system.

Moreover, the following inclusion criteria were applied: Male or female aged 18 years or older at screening, able to cooperate with consent procedures, able to participate in study activities including all required clinical assessments and biological donations. The exclusion criteria comprised the following: Unable to participate in consent procedures, current treatment with anti-coagulants, unable to participate in biological specimen collection due to a medical condition or medication status, parkinsonism of different nature than idiopathic, such as other forms of atypical or vascular parkinsonism and severe dementia.

The Ethics Committee for the Copenhagen Regional Area gave approval for this protocol (H-1-2011-093), and all participants gave written informed consent. For summarized patient and control demographic characteristics see Table 1.

**Table 1:** Demographic characteristics of control subjects, Parkinson’s Disease and Multiple System Atrophy patients

	MSA patients	PD patients	Normal controls
	*N* = 18	*N* = 46	*N* = 41
Age[years] (mean)	47–76 (62.5)	46–78 (62.4)	21–85 (43.9)
[SD]	[9.2]	[6.7]	[14.2] *P* < 0.001
Gender Female/Male	12/6	17/27	43/7 Chi-squared test: *p* < 0.0001
Age at onset [years] (mean)	42–75 (56.71)	37–74 (53.95)	
[SD]	[9.17]	[9.05]	
Disease duration [years] (mean)	1–10 (4.8)	1–22 (7.9)	-
[SD]	[2.6]	[5.0]	
Severity of the disease Hoehn and Yahr Staging (median)	2.0–5.0 (3.0)	1.5–2.5 (2.0) *P* < 0.0001	-

Demographic characteristics of control subjects, Parkinson’s Disease and Multiple System Atrophy patients

### Plasma samples

Venous blood was drawn at the respective clinics and processed on the same day at Bispebjerg *Movement Disorders* Biobank. All plasma samples were collected at inclusion. Samples were collected in EDTA coated polypropylene tubes, and were spun at 2000×g for 10 min at 4 °C; the supernatant plasma was then aliquoted and stored in 400 μL polypropylene (PP) tubes at −80 °C until the day of analysis, when they were thawed on ice for 30 min.

### Measurement of Anti-α-Synuclein Antibodies by Competitive ELISA

Levels and avidity of anti-α-synuclein NAbs were measured by competitive enzyme-linked immunosorbent assay (ELISA). 96-well polystyrene microtiter plates (Nunc MaxiSorp® flat-bottom 96 well plate) were coated with either 10 μg/mL or 5 μg/mL recombinant α-synuclein monomer (rPeptide, #S-10001-2) in ice-cold 0.1 M carbonate buffer (pH 8.5) overnight (>12 h) at 4 °C. The plates were emptied and blocked for 2 h at room temperature (RT) with 3% bovine serum albumin (BSA) and 0.1% NP-40 in phosphate-buffered saline (PBS, pH 7.4100 μL per well) and washed 5 times with PBS + 0.05% Tween-20. Next, 50 μL portions of plasma (diluted 1:100, 1:200, and 1:400 in PBS + 0.1%BSA) were transferred to the coated plates and incubated for 1 h at RT. For the competition reaction, plasma samples were incubated before transfer onto plates for 1 h with α-synuclein monomer at a range of concentrations: (1000, 250, 62.5, 15.5, 3.9, 0.97 and 0.24 nM for competition curve with plasma pools, and 1000, 50 and 2 nM for individual samples (the range of α-synuclein monomer concentrations was decided after completing preliminary experiments). After five washes with PBS + 0.05% Tween 20, 50 μL of peroxidase-labeled polyclonal goat anti-human IgG (Fc fragment specific; Abcam #ab98567: 1:20,000 dilution) was added to each well and incubated at RT for 2 h. After five more washes, tetramethylbenzidine (TMB) Liquid Peroxidase Substrate (50 μL; Sigma-Aldrich #T8665) was added, followed by incubation in the dark at RT for 30 min. The reaction was then stopped with 50 μL of 0.5 N H_2_SO_4_, and the absorbance at 450 nm was measured on a Fisher Scientific™ Multiskan™ FC Microplate Reader.

### Measurement of α-Synuclein Concentration by MSD Assay System

The α-synuclein concentration in plasma samples was measured with commercially available Human α-Synuclein Kit from MSD (Meso Scale Discovery®; MULTI-ARRAY Assay Systems, #K151TGD). The assay was performed as per the manufacturer’s instructions, with 1:50 dilution of plasma in Diluent-35, as provided by the manufacturer. For measurement of α-synuclein–anti-α-synuclein antibody complexes, samples were incubated with Sulfo-TAG anti-human IgG (MSD, #R32AJ-1). α-Synuclein/NAbs complexes were quantified with reference to an α-synuclein standard curve. The plates were read using an MSD Sector Imager S600 instrument, and the data analyzed using Discover Workbench 4.0 software.

### Measurement of Anti-α-Synuclein Antibodies by MSD Assay System

To validate our ELISA results we have optimized an MSD electrochemiluminescence assays to measure anti-α-synuclein NAbs in plasma samples. MSD assays present several advantages over traditional ELISAs, including increased sensitivity and low background [32]. 96-well microtiter plates (Standard MSD bind plate #L15XA-1) were coated with either 5 ng/mL or 0.5 ng/mL of recombinant α-synuclein monomer (rPeptide, #S-10001-2) in ice-cold 0.1 M carbonate buffer (pH 8.5) overnight (>12 h) at 4 °C. The plates were washed 3 times with washing buffer (PBS + 0.05% Tween 20) and blocked for 1 h at RT with 3% BSA fraction V and 0.1% NP-40 in PBS, pH 7.4, 150 μL per well on a shaker set at 800 rpm. The plates were washed 3 times with PBS + 0.05% Tween-20. The 25 μL portions of plasma (diluted 1:500 in PBS + 0.1%BSA fraction V) were transferred to the coated plates and incubated for 1 h at RT on the shaker. Before transfer onto plates, plasma pools were incubated for 1 h with α-synuclein monomer at a range of concentrations 8000, 2000, 500, 125, 31.2, 7.8, 1.95, 0.48, 0.122, 0.03 and 0.007 nM). The range of α-synuclein monomer and plasma concentrations were decided after preliminary experiments. Wells were then washed 3 times with PBS + 0.05% Tween 20, and 25 μL of MSD Sulfo Tag Goat anti-human IgG antibody (MSD #R32AJ-1; 1:500 dilution in PBS + 0.1% BSA fraction V) was added to each well, with incubation at RT for 1 h on the shaker. After three more washes, MSD Read Buffer at 1:2 concentration diluted in MilliQ water was added to the wells. Immediately afterwards, the plates were read using an MSD Sector Imager S600 instrument.

### Measurement of Anti-β/γ-Synuclein Antibodies by Competitive ELISA

Levels and avidity of anti-β/γ-synuclein NAbs were measured by competitive enzyme-linked immunosorbent assay (ELISA). 96-well polystyrene microtiter plates (Nunc MaxiSorp® flat-bottom 96 well plate) were coated with either 10 μg/mL recombinant β-synuclein monomer (rPeptide,GA,USA, #S-1003-2) or 10 μg/mL recombinant γ-synuclein monomer (rPeptide,GA,USA, #S-1007-1) in ice-cold 0.1 M carbonate buffer (pH 8.5) overnight (>12 h) at 4 °C. The plates were emptied and blocked for 2 h at room temperature (RT) with 3% bovine serum albumin (BSA) and 0.1%NP-40 in phosphate-buffered saline (PBS, pH 7.4, 100 μL per well) and washed 5 times with PBS + 0.05% Tween-20. Fifty μL portions of plasma diluted 1:400 in PBS + 0.1%BSA were transferred to the coated plates and incubated for 1 h at RT. For the competition reaction, before transfer onto plates, samples were incubated for 1 h with either β-synuclein or γ-synuclein monomer at a 2-fold dilution range of concentrations: 1000-2 nM for competition curve with plasma pools, and 1000, 100 and 10 nM for individual samples (the range was decided after completing preliminary experiments). After five washes with PBS + 0.05% Tween-20, 50 μL of peroxidase-labeled polyclonal goat anti-human IgG (Abcam, UK: 1:20,000 dilution) was added to each well and incubated at RT for 2 h. After five more washes, tetramethylbenzidine (TMB) Substrate (50 μL; Sigma-Aldrich,MO,USA) was added. After 30 min the reaction was then stopped with 50 μL of 0.5 N H2SO4, and the absorbance at 450 nm was measured on a Fisher Scientific™ Multiskan™ FC Microplate Reader,MA,USA.

### In vitro phosphorylation of human α-synuclein

Alpha-synuclein (Abcam #ab51189) was phosphorylated using PLK 2 (Invitrogen Cat# PV4204) at a concentration of 1.44 mg/ml (100 μM). The phosphorylation reactions were carried out in the presence of 1.09 mM ATP, 1× reaction solution (2 mM HEPES, 10 mM MgCl_2_, 2 mM DDT, pH 7.4) and 1 μg of PLK/144 μg/ml of α-syn at 30 °C for 24 h. The reaction was quenched with 25 mM EDTA. After quenching the sample was desalted on a G25 column (HiTrap Desalting, GE healthcare #G-25 17–1408-01) into DPBS (Invitrogen #14190–094).

The sample was analyzed by LC-MS. Briefly; the sample was separated on a C4 2.1 × 50 mm BEH300 column run in FA/ACN and introduced to a XEVO QTOF Mass spectrometer (Waters). The multicharged signal obtained from the ion trace was deconvoluted and the mass identified to be a mixture of a non-phosphorylated 14,459 Da α-synuclein (minor component) and the major 14,539 Da phosphorylated species corresponding to a + 80 Da change caused by phosphorylation on serine 129.

### Measurement of Anti-Phosporylated-α-Synuclein Antibodies by Competitive MSD Assay System

We have optimized an MSD electrochemiluminescence assays to measure binding properties of anti-P-α-synuclein NAbs in plasma samples. 96-well microtiter plates (Standard MSD bind plate #L15XA-1) were coated with either 5 ng/mL recombinant α-synuclein monomer (rPeptide, #S-10001-2) or 5 ng/mL P-α-synuclein in ice-cold 0.1 M carbonate buffer (pH 8.5) overnight (>12 h) at 4 °C. The plates were washed 3 times with washing buffer (PBS + 0.05% Tween 20) and blocked for 1 h at RT with 3% BSA fraction V and 0.1% NP-40 in PBS, pH 7.4, 150 μL per well on a shaker set at 800 rpm. The plates were washed 3 times with PBS + 0.05% Tween-20. The 25 μL portions of plasma (diluted 1:400 in PBS + 0.1%BSA fraction V) were transferred to the coated plates and incubated for 1 h at RT on the shaker. Before transfer onto plates, plasma samples were incubated for 1 h with α-synuclein monomer at 1 μM and a range of P-α-synuclein concentrations 10, 1, and 0.1 nM. For competition curve, plasma pools (diluted 1:400 in PBS + 0.1%BSA fraction V) were incubated for 1 h with P-α-synuclein at a 3-fold dilution range of concentrations 100 nM-0.3pM. The range of P-α-synuclein and plasma concentrations were decided after preliminary experiments.

Wells were then washed 3 times with PBS + 0.05% Tween 20, and 25 μL of MSD Sulfo Tag Goat anti-human IgG antibody (MSD #R32AJ-1; 1:500 dilution in PBS + 0.1% BSA fraction V) was added to each well, with incubation at RT for 1 h on the shaker. After three more washes, MSD Read Buffer at 1:2 concentration diluted in MilliQ water was added to the wells. Immediately afterwards, the plates were read using an MSD Sector Imager S600 instrument.

### Cross-binding inhibition assays

To obtain the two site inhibition curves, ten plasma samples from each group were pooled and incubated in 1:400 dilution with increasing concentration of α-, β-, and γ-synuclein monomers at a 2-fold dilution range of concentrations 1000-2 nM, in combinations: α- and β-synuclein; α- and γ-synuclein, β- and γ-synuclein, with subsequent measurement of free NAbs by ELISA (as described above) on plates coated with 10 μg/ml of α-synuclein. For individual samples (the range was decided after completing preliminary experiments) 1000, 100 and 10 nM concentrations of α-, β-, and γ-synuclein monomers were used in the same combinations as for the inhibition curves. To obtain relative maximum of inhibition, each sample has been preincubated with high concentration (1000 nM) of mixed together α-, β-, and γ-synuclein.

### Sample analyses

The relative binding of anti-α-, β-, and γ-synuclein NAbs is expressed as a percentage of maximum binding observed in the assay for each sample; the competition reactions with 1000 nM α-, β- or γ-synuclein monomer were defined as representing 0% binding (unspecific binding), and reactions without competition are taken to indicate 100% (maximum) of binding in the displacement curves. For the inhibition assays, results are expressed as % inhibition calculated as [(OD_450_ of non-inhibited sample) ÷ (OD_450_ of inhibited sample)] × 100/OD_450_ of non-inhibited sample, where OD_450_ is the optical density at 450 nm.

Due to unequal group sizes, non-normal distribution of some variables, and rank-order ratings on the Hoehn and Yahr staging scale, non-parametric tests (The Kruskal–Wallis one-way analysis of variance, Mann–Whitney U Test and Spearman correlation) were used for group comparisons and correlation analyses. All tests were carried out using GraphPad Prism software v.6 (GraphPad Software Inc., La Jolla, CA). Differences were considered significant at the *P* value of less than 0.05.

Analysis of the competition binding curves was performed according to the one- and two-site models using computer-assisted curve fitting Fit logIC50 model (GraphPad Software Inc., La Jolla, CA)

## Results

### Pre-analytical evaluations of competitive ELISA set-ups

**Fig. 1:**
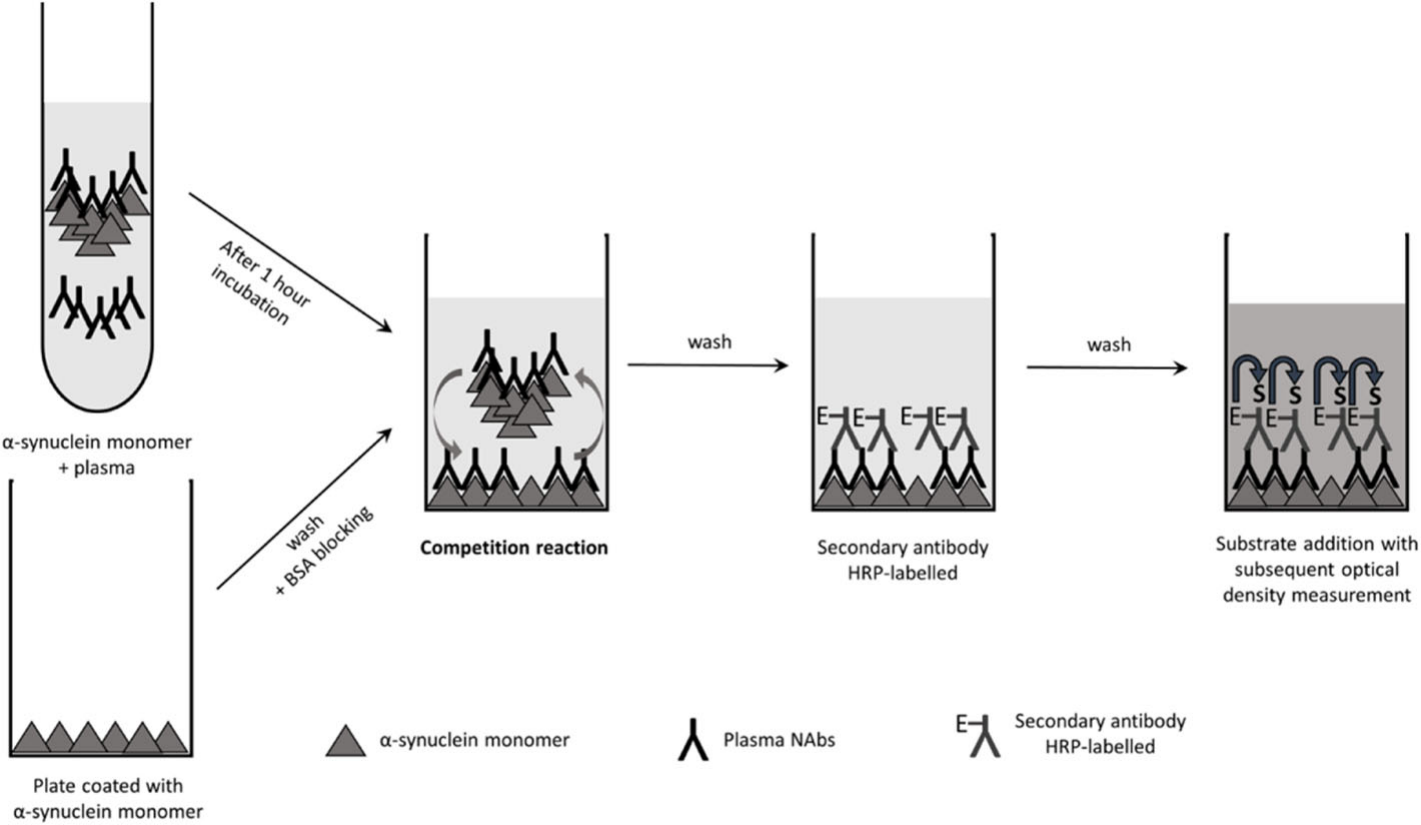
Schematic overview of the competitive ELISA technique. Before transfer onto plates coated with α-synuclein monomer, plasma samples are incubated for 1 h at RT with α-synuclein monomer. Following washing steps, the competition reaction is created by moving plasma/α-synuclein monomer samples onto the coated plate. Here, high affinity antibodies form more stable bonds with free antigen than do lower affinity antibodies. Consequently, lower affinity antibodies remain bound to the excess immobilized antigen on the plates, but high-affinity antibodies bound to the fluid-phase antigen are washed away. After several washes, the amounts of low-affinity antibodies remaining on the plates are detected by peroxidase-labelled polyclonal goat anti-human IgG (Fc fragment specific). The plate is developed by adding an enzymatic substrate to produce a visible signal

Polyclonal plasma antibodies against a particular antigen potentially comprise a mixture of antibodies with different epitope specificities and affinities. We employed the competitive ELISA method to measure relative binding properties of the composite of anti-α-synuclein NAbs in plasma, exploiting the stronger antibody-antigen interactions of high affinity antibodies. By incubating plasma samples with increasing concentrations of free α-synuclein monomer prior to measurement of free antibody titre on plates with immobilized α-synuclein monomer we were able to separate anti-α-synuclein NAbs into a low and high affinity/avidity pools. The schematic diagram of the assay setup is presented in Fig. 1.

**Fig. 2:**
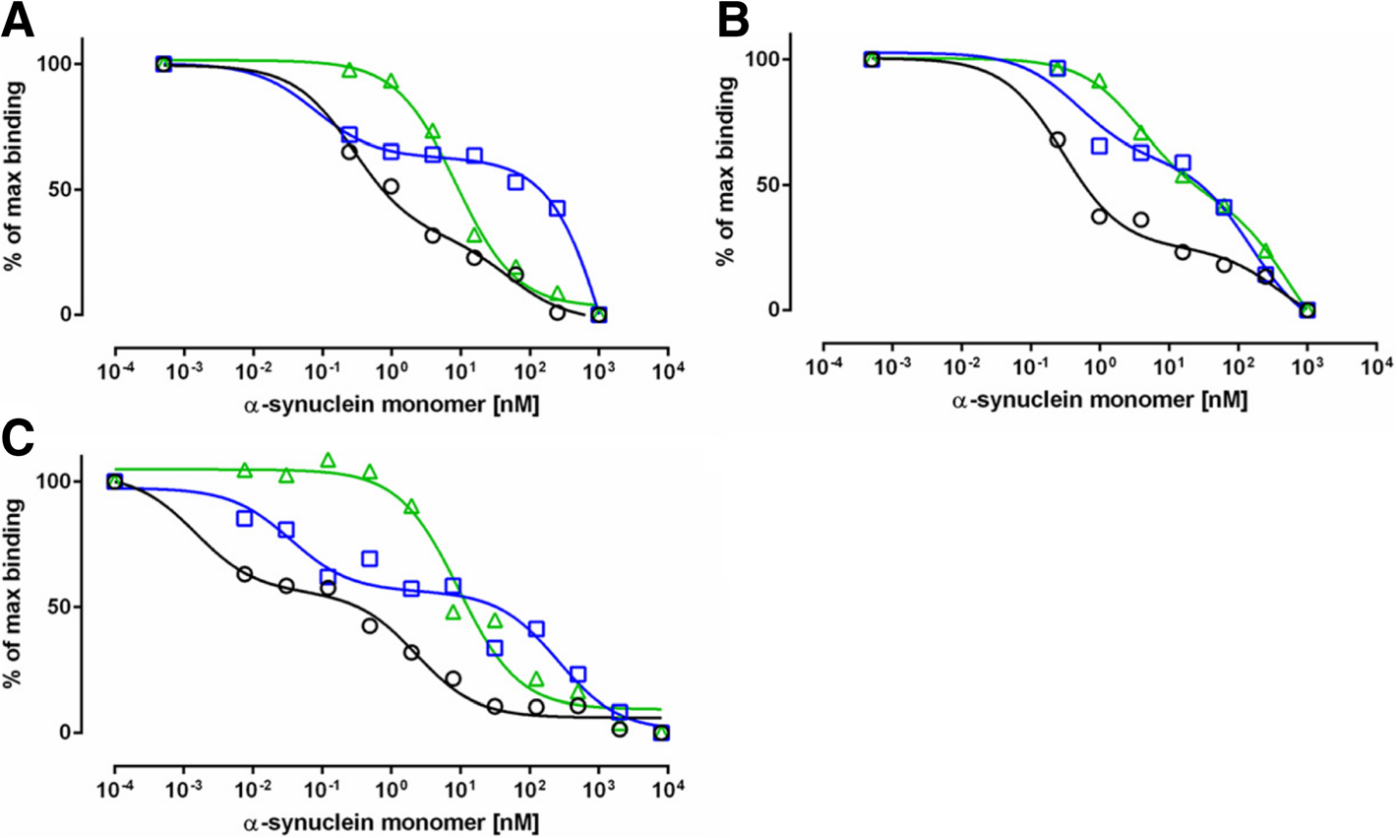
The two site inhibition curves show distinct high and low binding components in plasma from Parkinson’s Disease (*PD-blue, squares*), Multiple System Atrophy (*MSA-green, triangles*) patients and normal control (*NC-black, circles*) subjects. Ten plasma samples from each group were pooled and incubated with increasing concentration of α-synuclein monomer with subsequent measurement of free NAbs by ELISA or Meso Scale Discovery (MSD) assays. **a** ELISA: 10 μg/mL α-synuclein coating, plasma dilution 1:400; **b** ELISA: 5 μg/mL α-synuclein coating, plasma dilution 1:200; **c** MSD assay: 5 ng/mL α-synuclein coating, plasma dilution 1:500. The relative binding of anti-α-synuclein NAbs is expressed as a percentage of maximal binding attained in the assay for each sample, where competition reactions with 1000 nM α-synuclein monomer are defined as constituting non-specific (background) binding and reactions without competition reflect 100% (maximum) binding. The line represents the fitting of a two site model to the data

For the initial series of experiments, 10 randomly selected plasma samples from each group were pooled. The pooled samples were used to determine the appropriate plasma dilutions suitable for inhibition assays, as well as the concentrations of the coating antigen required to test the individual samples in the later part of the study. Fig. 2a, b present examples of the binding curves obtained in competitive ELISA, showing distinct apparent affinity profiles of anti-α-synuclein plasma NAbs from PD, MSA patients and normal controls. These displacement curves are expressed as percentage of the maximal binding measured in the absence of free α-synuclein monomers. High affinity/avidity is indicated by efficient inhibition of antibody binding in the presence of low (1–10 nM) concentrations of α-synuclein, whereas low affinity/avidity binding type is revealed by inhibition at a high concentration (10–1000 nM). The inhibition curve distinctly follows a two-site model for plasma from normal controls, indicating that a substantial fraction (approximately 50%) of total antibody binding is characterized by antibodies with high apparent affinity/avidity. The remaining part of total antibody binding indicates antibodies of low affinity, as well as the contribution of unspecific binding. Displacement curves from PD patient plasma samples also supported a two-affinity state model but, with a reduced fraction of high affinity antibodies. In contrast, the MSA plasma required high α-synuclein concentration for inhibition indicating an absence of high affinity antibodies. These results were further supported by using the MSD assay platform. By applying the MSD assay we were able to reduce the concentration of coating antigen from 10 μg/mL and 5 μg/mL to 5 ng/mL (Fig. 2c) and 0.5 ng/ml (not shown), due to sensitivity of the assay, and to test the pooled plasma samples at 1:500 dilutions. The binding curves obtained by MSD were in accordance with those obtained using ELISA, in that we could further discern the two site binding model comprising a high affinity component at low nM range and a low affinity group with approximate affinity around 1 μM.

**Fig. 3:**
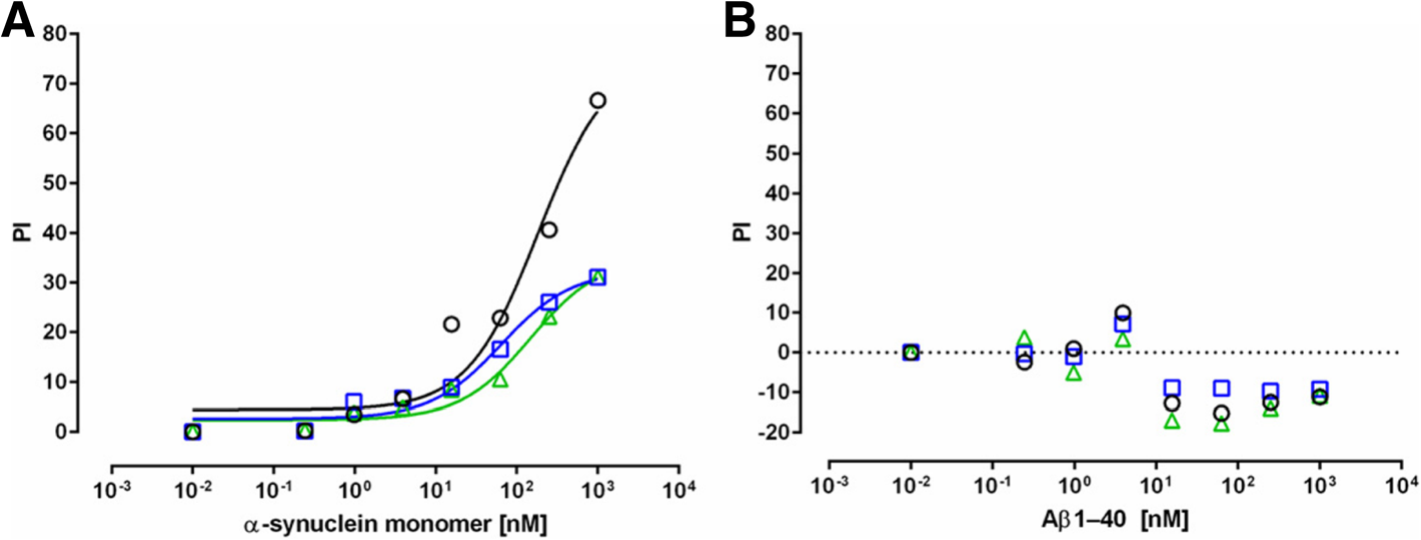
Specificity of anti-alpha-synuclein NAb binding. The ability of increasing concentrations of **a** α-synuclein monomer or **b** amyloidβ1–40 (Aβ1–40) to inhibit the binding of α-synuclein NAbs to α-synuclein coated ELISA plates. Data are presented as % inhibition (PI) relative to the unblocked condition. Pooled plasma samples are from 10 Parkinson’s Disease (*PD-blue, squares*), 10 Multiple System Atrophy (*MSA-green, triangles*) patients and 10 normal controls (*NC-black, circles*) were diluted 1:400. The lines in **a** represents the fitting of a one site model to the data

To evaluate the specificity of the binding of anti-α-synuclein plasma NAbs, control competition assays were conducted on the pooled plasma samples using antigens unrelated to synucleinopathies. Here we performed ELISA assay using either α-synuclein monomer or amyloid β1–40 (Aβ1–40) peptides in the fluid inhibition phase and plates coated with α-synuclein at 5μg/mL (Figs. 3a, b). The α-synuclein monomer were able to inhibit plasma NAbs binding in all tested groups (Fig. 3a), whereas there was no inhibition detected with Aβ1–40 (Fig. 3b) suggesting the specificity of anti-α-synuclein plasma NAbs.

### Binding properties of anti-α synuclein NAbs

**Fig. 4:**
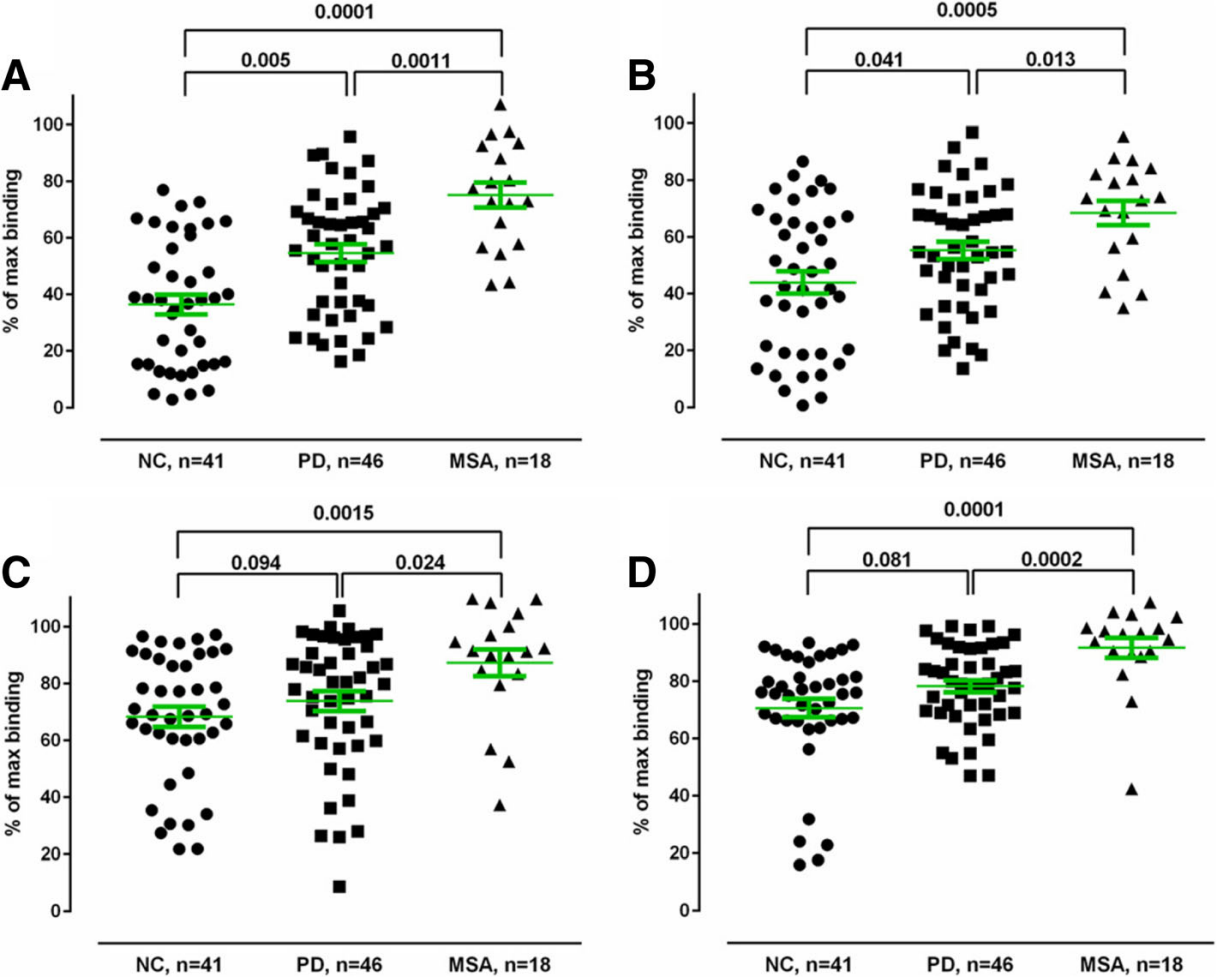
Reduced levels of high affinity anti-α-synuclein NAbs in plasma from MSA and PD patients. Binding of anti-α-synuclein NAbs in plasma to immobilized α-synuclein monomer (5 μg/mL) in competitive ELISA assay in the presence of **a**, **b** 50 nM or **c**, **d** 2 nM free α-synuclein. Non-specific binding is defined as the binding to the plate in the presence of 1000 nM free α-synuclein monomer. Maximal binding is defined as the binding observed in the absence of added free α-synuclein monomer. Horizontal bars represent the mean values +/− SEM. Plasma samples from Parkinson’s Disease (PD), Multiple System Atrophy (MSA) patients and normal controls (NC) were tested at two dilutions, i.e. **a**, **c** 1:100 and **b**, **d** 1:200. Significance was tested using Man–Whitney’s U test (*P* < 0.05)

Consequently, we proceeded with the ELISA set-up to test the binding properties of the anti-α-synuclein NAbs in the individual plasma samples from all 46 PD and 18 MSA patients, and the 41 controls. . Each plasma sample was diluted 1:100 and 1:200 with PBS and preincubated with low 2 nM, 50 nM (Fig. 4a–d respectively) and high concentrations (1 μM) of free α-synuclein monomer to saturate the inhibition reaction, followed by incubation on plates coated with 5 μg/ml of immobilized α-synuclein. The data show that in the presence of 50 nM free α-synuclein monomer a binding of approximately 70–80% of the maximal binding is obtained for anti-α-synuclein NAbs in plasma from MSA patients. The corresponding values for PD and normal controls are significantly lower 50–55% and 40–45% respectively (Fig. 4a–b), indicating that these plasma samples contain a significant larger proportion of high affinity anti-α-synuclein NAbs compared to MSA patients. In the same experiment performed in the presence of 2 nM free α-synuclein monomer a binding of approximately 90–95% of the maximal binding is obtained for anti-α-synuclein NAbs in plasma from MSA patients. The corresponding values for PD and normal controls are approx. 80% and 70–75% respectively, (Fig. 4c-d). Under these conditions both MSA and PD are significant different from healthy controls, meaning that both MSA and PD patients contains a significant lower amount of high affinity anti-α-synuclein NAbs in plasma compared to controls. These are the key findings in the study.

**Fig. 5:**
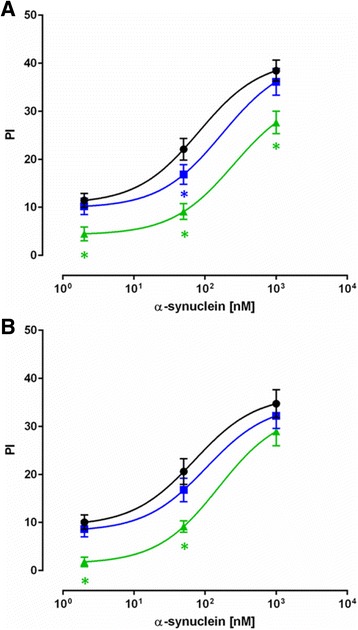
Plasma samples from MSA patients have significantly reduced binding capability to α-synuclein monomer. Average percentage (± − SEM) of inhibition (PI) of individual plasma samples with free α-synuclein monomer on plates coated with 5 μg/mL of α-synuclein monomer. Plasma samples from Parkinson’s Disease (*PD-blue squares*), Multiple System Atrophy (*MSA-green, triangles*) patients and normal controls (*NC-black, circles*) were tested at two different dilutions **a** 1:100 and **b** 1:200. The line represents the fitting of a two-site model to the data (*p < 0.05)

We furthermore tested the ability of α-synuclein monomer to inhibit binding to α-synuclein coated plates of NAbs from individual samples, using the same conditions as described for maximal binding in the experiments above. We expressed the percent inhibition relative to that in the unblocked plasma, i.e. samples with no free antigen added. Both 1:100 (Fig. 5a) and 1:200 (Fig. 5b) diluted plasma samples from MSA patients had significantly reduced binding capability for α-synuclein monomer in comparison with plasma samples from PD patients and healthy controls. Only with a low plasma concentration (1:200) and high antigen levels (1 μM) could we achieve comparable inhibition in all groups (Fig. 5b). This further supports our finding of absence of high affinity anti-α-synuclein NAbs in plasma samples from MSA patients.

### Effect of age

**Fig. 6:**
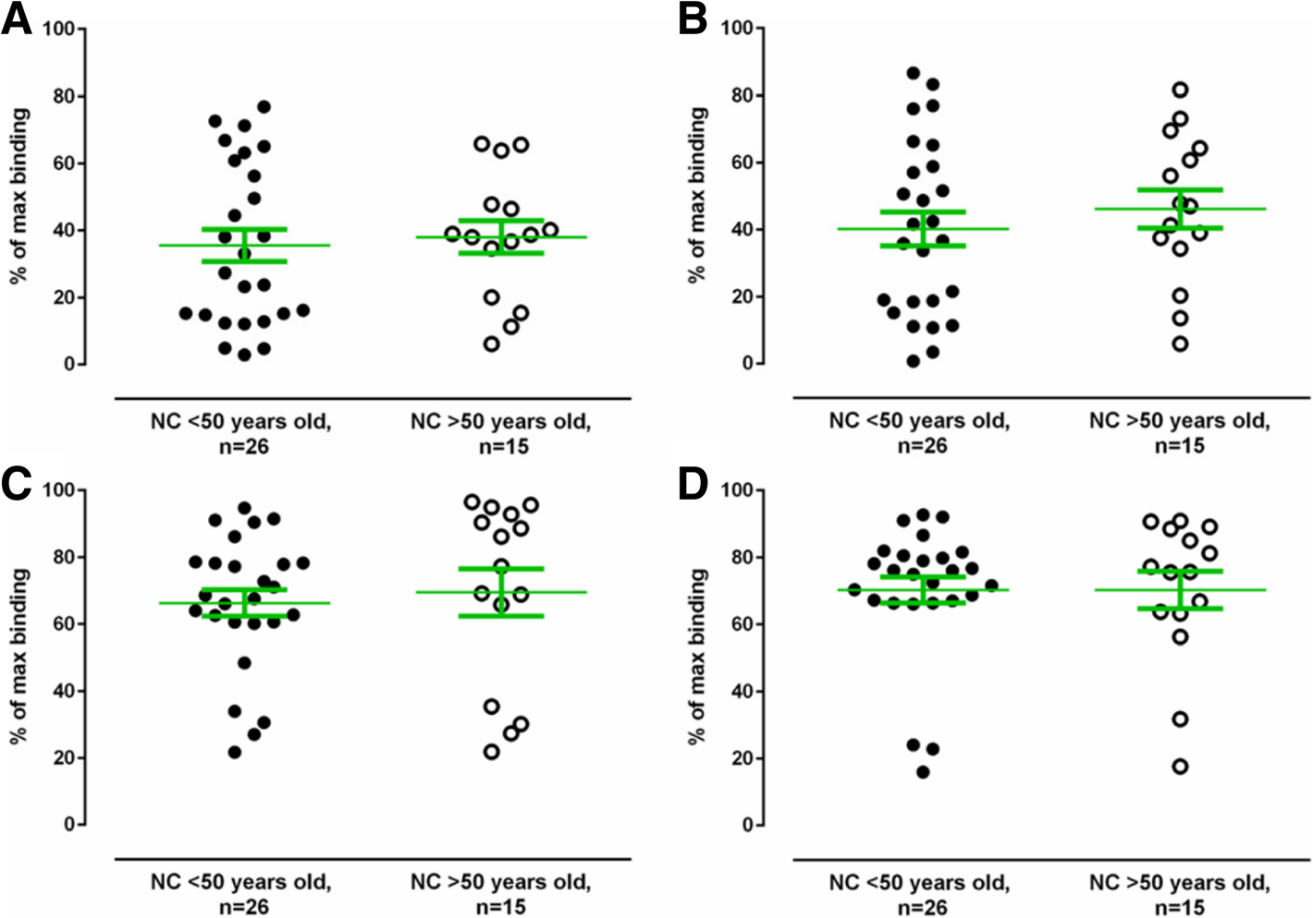
Absent correlation of age and binding properties of anti-α-synuclein NAbs in plasma from negative controls. Percentage of maximal plasma NAbs binding to immobilized α-synuclein monomer (5 μg/mL) in younger controls (<50 years old, *n* = 26) and in elderly controls (>50 years old, *n* = 15) determined by competitive ELISA assay with **a**, **b** 50 nM or **c**, **d** 2 nM of free α-synuclein, where 10,000 nM defines the non-specific binding and no free α-synuclein monomer gives the maximal binding. Horizontal bars represent the mean values ± SEM. Plasma samples were diluted tested in two different dilutions: **a**, **c** 1:100 and **b**, **d** 1:200

There is evidence suggesting that the general changes in the humoral immune responses with age are qualitative rather than quantitative, i.e. it is the affinity and specificity of the antibodies that change, rather than the quantity of antibodies produced [33]. Due to the difference in mean age of our control subjects (Table 1), we tested if the maximal binding to α-synuclein of NAbs from elderly controls (>50 years old) differed from that of younger controls (<50 years old). There was no significant difference in binding properties of anti-α-synuclein NAbs from plasma of controls stratified by age, either at a dilution of 1:100 (Fig. 6a, b) or 1:200 (Fig. 6d, c).

### Levels of plasma α-synuclein and α-synuclein/NAbs complexes

**Fig. 7:**
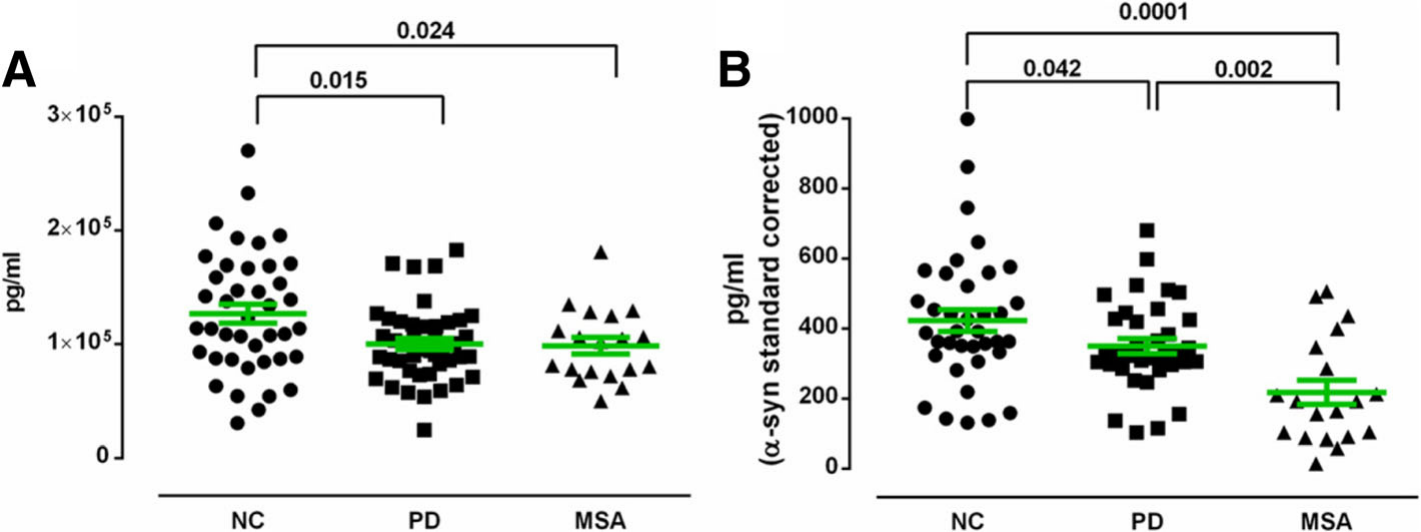
Concentrations of α-synuclein **a** and α-synuclein-NAbs complexes **b** in plasma from Parkinson’s Disease (PD), Multiple System Atrophy (MSA) patients and normal controls (NC). Horizontal bars represent the mean values ±SEM. α-Synuclein-NAbs complex concentrations were quantified using α-synuclein standard curve. Significance was tested using Man–Whitney’s U test (*P* < 0.05)

In addition to measuring the individual binding properties of the plasma NAbs, we also measured the concentration of plasma α-synuclein and α-synuclein/NAbs complexes. The mean (±SD) plasma concentration of α-synuclein was significantly higher in the control group (127 ± 8.3 ng/ml) than for PD (100.2 ± 3.2 ng/ml; *p* = 0.015) and MSA (98.6 ± 3.1 ng/ml; *p* = 0.024) patients (Fig. 7a). To measure α-synuclein/NAbs complexes in plasma, the α-synuclein detection antibody in the human α-synuclein MSD kit had been substituted with an equivalent amount of an anti-human IgG antibody, while the nature of the capture antibody was unchanged. The results here show a significantly higher α-synuclein/NAbs complex concentration in controls (423 ± 31.2 pg/ml) than in PD (350 ± 21.6 pg/ml; *p* = 0.042) and MSA (217 ± 34.7 pg/ml; *p* = 0.0001) patients (Fig. 7b). The plasma α-synuclein/NAbs complex concentration was significantly lower in MSA than PD patients (*p* = 0.002).

### Binding properties of anti-β-synuclein and anti-γ-synuclein NAbs

**Fig. 8:**
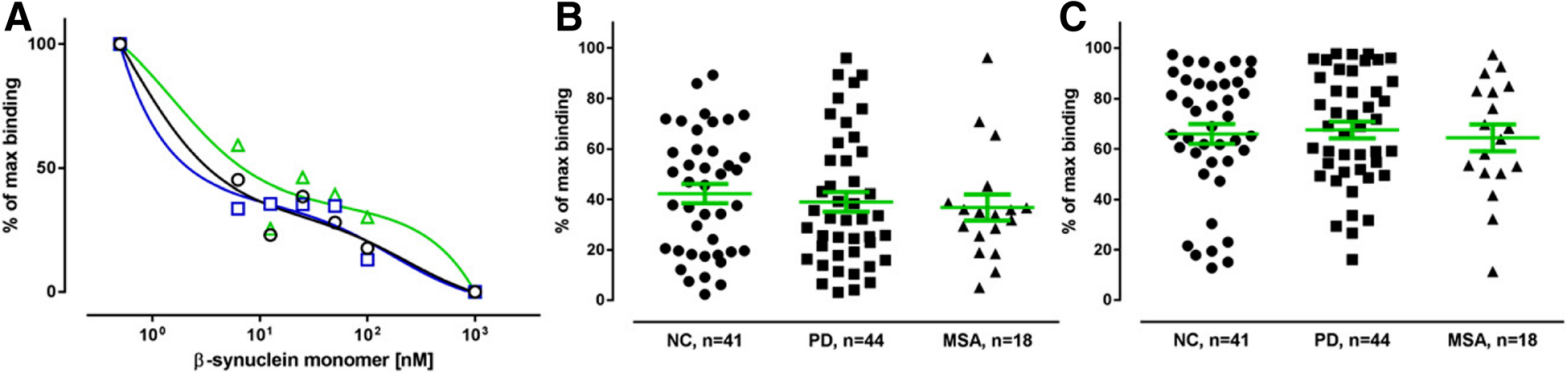
β-synuclein competition ELISA assays. **a** The two site inhibition curves show high and low binding components in pooled plasma samples from Parkinson’s Disease (*PD-blue, squares*), Multiple System Atrophy (*MSA-green, triangles*) patients and normal control (*NC-black, circles*) subjects. 10 μg/mL β-synuclein coating, plasma dilution 1:400; **b** and **c**: Binding of anti-β-synuclein NAbs in individual plasma samples to immobilized β-synuclein monomer (10 μg/mL) in competitive ELISA assay in the presence of **b** 100 nM or **c** 10 nM free β-synuclein. Non-specific binding is defined as the binding to the plate in the presence of 1000 nM free β-synuclein monomer. Maximal binding is defined as the binding observed in the absence of added free β-synuclein monomer. Plasma samples were tested at 1:400 dilution. Horizontal bars represent the mean values +/− SEM. Significance was tested using Man–Whitney’s U test (*P* < 0.05)

**Fig. 9:**
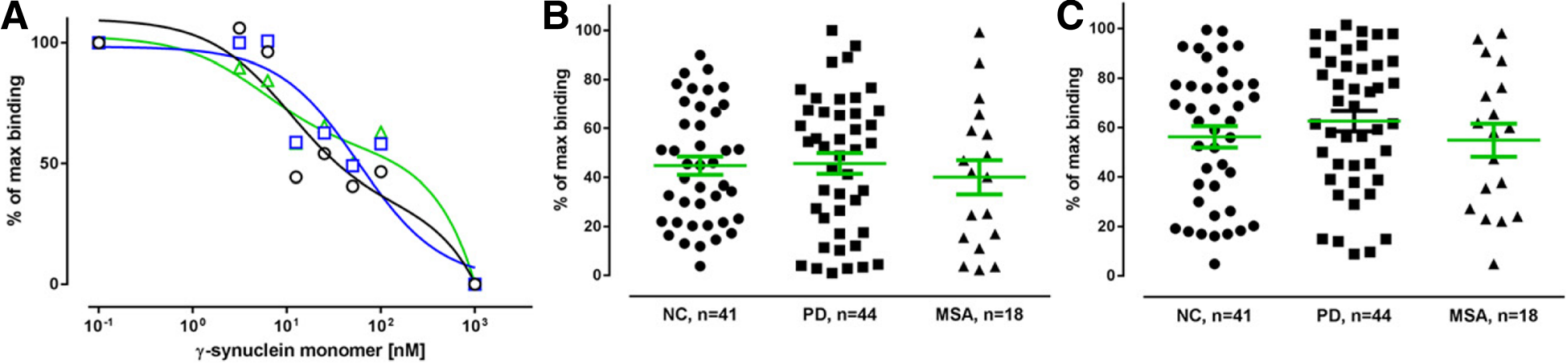
γ-synuclein competition ELISA assays. **a** The two site inhibition curves show high and low binding components in pooled plasma samples from Parkinson’s Disease (*PD-blue, squares*), Multiple System Atrophy (*MSA-green, triangles*) patients and normal control (*NC-black, circles*) subjects. 10 μg/mL γ-synuclein coating, plasma dilution 1:400; **b** and **c**: Binding of anti-γ-synuclein NAbs in individual plasma samples to immobilized γ-synuclein monomer (10 μg/mL) in competitive ELISA assay in the presence of **b** 100 nM or **c** 10 nM free γ-synuclein. Non-specific binding is defined as the binding to the plate in the presence of 1000 nM free γ-synuclein monomer. Maximal binding is defined as the binding observed in the absence of added free γ-synuclein monomer. Plasma samples were tested at 1:400 dilution. Horizontal bars represent the mean values +/− SEM. Significance was tested using Man–Whitney’s U test (*P* < 0.05)

The synuclein family consists of α-, β-, and γ-synucleins, that are 55–62% identical in sequence with most of the similarity lying within the N-terminus of the proteins (aa 1–100) and with more variability in sequence located toward the C-terminus. Because NAbs are often polyreactive, and may exhibit cross-reactivity, we tested if the binding properties of the anti-α-synuclein NAbs would be reflected in anti-β and -γ-synuclein NAbs. Figures. 8a and 9a present examples of the binding curves obtained in competitive ELISA on pooled samples (*n* = 10) for each disease and controls, showing similar apparent affinity profiles of anti-β-synuclein and anti-γ-synuclein, respectively, for plasma NAbs from PD, MSA patients and normal controls. Further, we proceeded with the ELISA set-up to test the binding properties of the anti-β and anti-γ-synuclein NAbs in the individual plasma samples from all 46 PD and 18 MSA patients, and the 41 controls. Each plasma sample was diluted 1:400 with PBS and preincubated with low (10 nM and 100 nM) and high concentrations (1 μM) of either free β-synuclein (Fig. 8b, c) or γ-synuclein monomer (Fig. 9b, c), followed by incubation on plates coated with 10 μg/ml of immobilized β- or γ-synuclein respectively. The data show that in the presence of 10 nM free β- or γ-synuclein monomer a binding of approximately 50–70% of the maximal binding is obtained for anti-β- and anti-γ-synuclein NAbs in plasma from all three groups. The corresponding values for 100 nM free β- or γ-synuclein monomer are also similar in all tested groups and are in the range of 40–45%. These data suggest that there are no differences in the levels of high and low affinity components of anti-β- and anti-γ-synuclein NABs in plasma from PD, MSA patients and normal controls.

### Cross-binding experiments

**Fig. 10:**
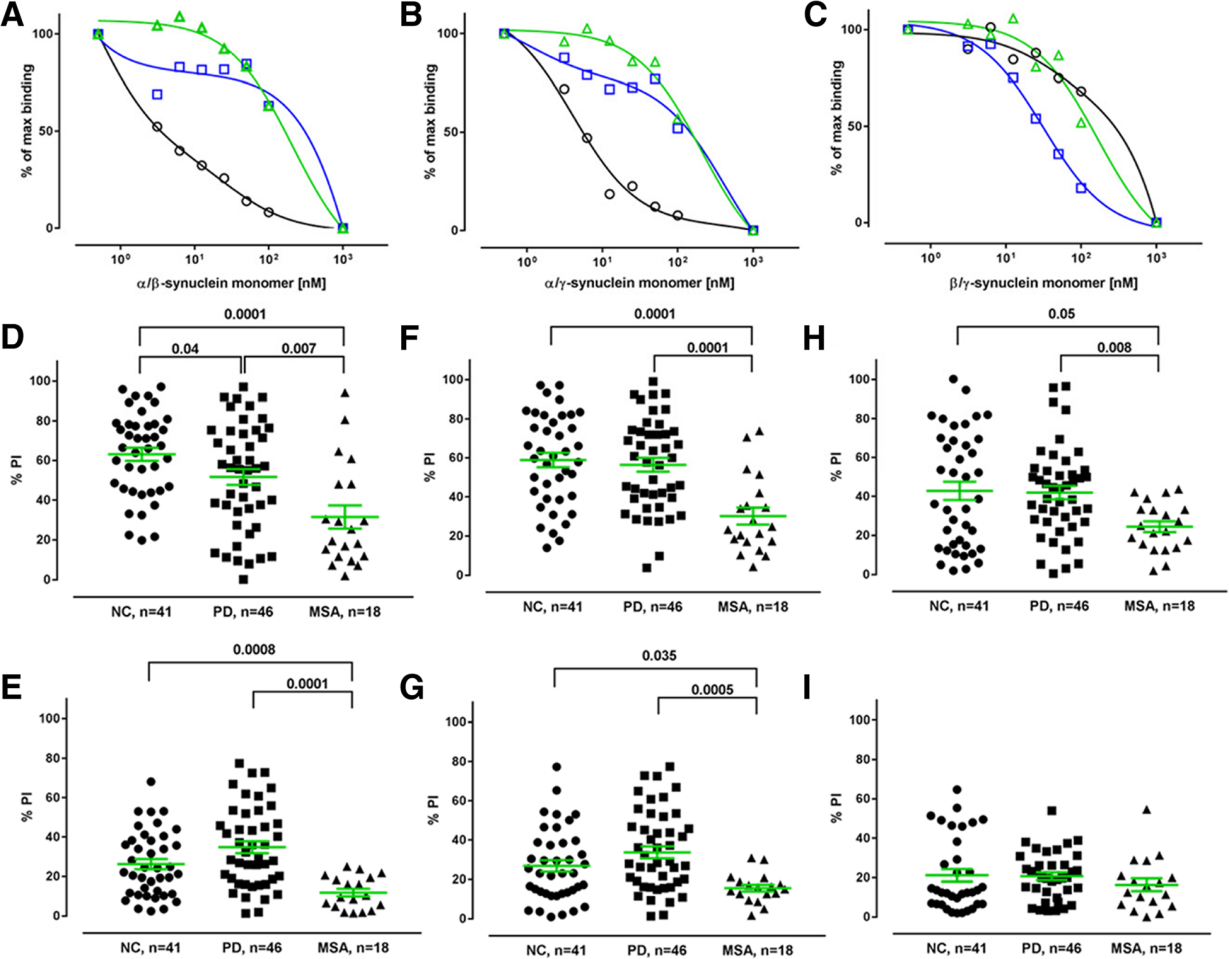
Cross-binding competition ELISA assays. **a**, **b**, and **c**: The two site inhibition curves show distinct high and low binding components in plasma from Parkinson’s Disease (*PD-blue, squares*), Multiple System Atrophy (*MSA-green, triangles*) patients and normal control (*NC-black, circles*) subjects. Ten plasma samples from each group were pooled and incubated in 1:400 dilution with increasing concentration of α-, β-, and γ-synuclein monomers in combinations as indicated, with subsequent measurement of free NAbs by ELISA on plates coated with 10 μg/ml of α-synuclein. **d**-**i**: Percentage of inhibition (PI) of individual plasma samples with free **d**, **e**: α−/β-synuclein monomers, **F**, **G**: α−/γ-synuclein monomers; **h**, **i**: β−/γ-synuclein monomer on plates coated with 10 μg/mL of α-synuclein monomer. Plasma samples from Parkinson’s Disease (PD), Multiple System Atrophy (MSA) patients and normal controls (NC) were tested at 1:400 dilution in the presence of (**d**, **f**, **h**) 100 nM or (**e**, **g**, **i**) 10 nM α-, β-, and γ-synuclein monomers in combinations. Horizontal bars represent the mean values +/− SEM. Significance was tested using Man–Whitney’s U test (*P* < 0.05)

To evaluate cross-reactivity of anti-α-synuclein high and low affinity plasma NAbs with β- and γ-synuclein monomers, we have performed cross-binding ELISA set-ups. The inhibition curves for the scenario where α-synuclein is present simultaneously with either β- or γ synuclein distinctly follows a two-site model seen before with free α-synuclein (Fig. 10a and b). Accordant to the α-synuclein experiments plasma from normal controls, is characterized by antibodies with high apparent affinity/avidity. PD plasma samples also supported a two-affinity state model, but with a reduced fraction of high affinity antibodies, and to a lesser extent in comparison to when α-synuclein was present alone. MSA plasma samples required high α−/β-synuclein and α−/γ-synuclein concentrations for inhibition indicating an absence of high affinity antibodies. In the simultaneous presence of increasing concentrations of free β- and γ-synuclein monomers prior to measurement of free antibody titre on plates coated with α-synuclein monomer (Fig. 10c) plasma samples from normal individuals still supported a two-affinity state model but to a lower extend than in the previous set-ups, whereas plasma samples from PD and MSA patients required high β−/γ-synuclein concentrations for maximum of inhibition. These data indicate that anti-α-synuclein NAbs from normal controls cross-react with β- and γ-synuclein monomers whereas such cross-binding is not found in PD and MSA patients.

Subsequently, each individual plasma sample was diluted 1:400 with PBS and preincubated with low (10 nM or 100 nM) and high concentrations (1 μM) of free α-, β-, and γ-synuclein monomers in combinations analogous to the inhibition curve experiments, followed by incubation on plates coated with 10 μg/ml of immobilized α-synuclein. The data are presented as percent of inhibition relative to 100% where high levels (1 μM) of each α-, β-, and γ-synuclein were added simultaneously to the reaction, and to the unblocked plasma, i.e. samples with no free antigen added. The data show that in the presence of 100 nM free proteins an inhibition of approximately 60–70% is obtained for combinations α−/β-synuclein and α−/γ synuclein in plasma from normal controls. The corresponding values for PD and MSA patients are significantly lower 50% and 30% respectively for α−/β-synuclein (Fig. 10d), and 60% and 30% respectively for α−/γ synuclein. Notably in the presence of α−/γ synuclein we did not observe significant differences between healthy individuals and PD patients. In the presence of 10 nM concentrations of free α−/β-synuclein (Fig. 10e) and α−/γ synuclein (Fig. 10g) significant differences in the percentage of inhibition were observed in plasma from controls and PD patients vs. MSA patients, whereas no difference was found between PD patients and control individuals.

The inhibition levels of anti-α-synuclein NAbs by simultaneous presence of 100 nM β- and γ-synuclein monomers were decreased in comparison to the previous approach in normal controls and PD patients (approximately 40–45%), but still significantly higher than in MSA patients (Fig. 10h). No differences between the groups were observed with 10 nM of free β- and γ-synuclein monomers (Fig. 10i).

These data suggest the high affinity anti-α-synuclein plasma components, seen in healthy individuals, are selective for physiologically relevant epitopes and probably react solely with C-terminal epitopes of α-synuclein.

### Binding properties of anti-P-α-synuclein NAbs

Posttranslational modifications of α-synuclein, particularly phosphorylation at serine 129, may be critical in pathogenesis of PD and MSA [34]. The neuropathological diagnosis of PD and MSA is often based on specific regional distribution of P-α-synuclein in glial cells and neurons of the CNS, respectively. Therefore, we tested if the binding properties of the anti-α-synuclein NAbs would also be reflected in anti-P-α-synuclein NAbs. For that, an α-synuclein monomer preparation was phosphorylated with a serine⁄threonine kinase PLK2. PLK2 is a principle contributor to α-synuclein phosphorylation at Ser-129 in neurons and directly phosphorylates α-synuclein at Ser-129 in in vitro biochemical assays [35].

**Fig. 11:**
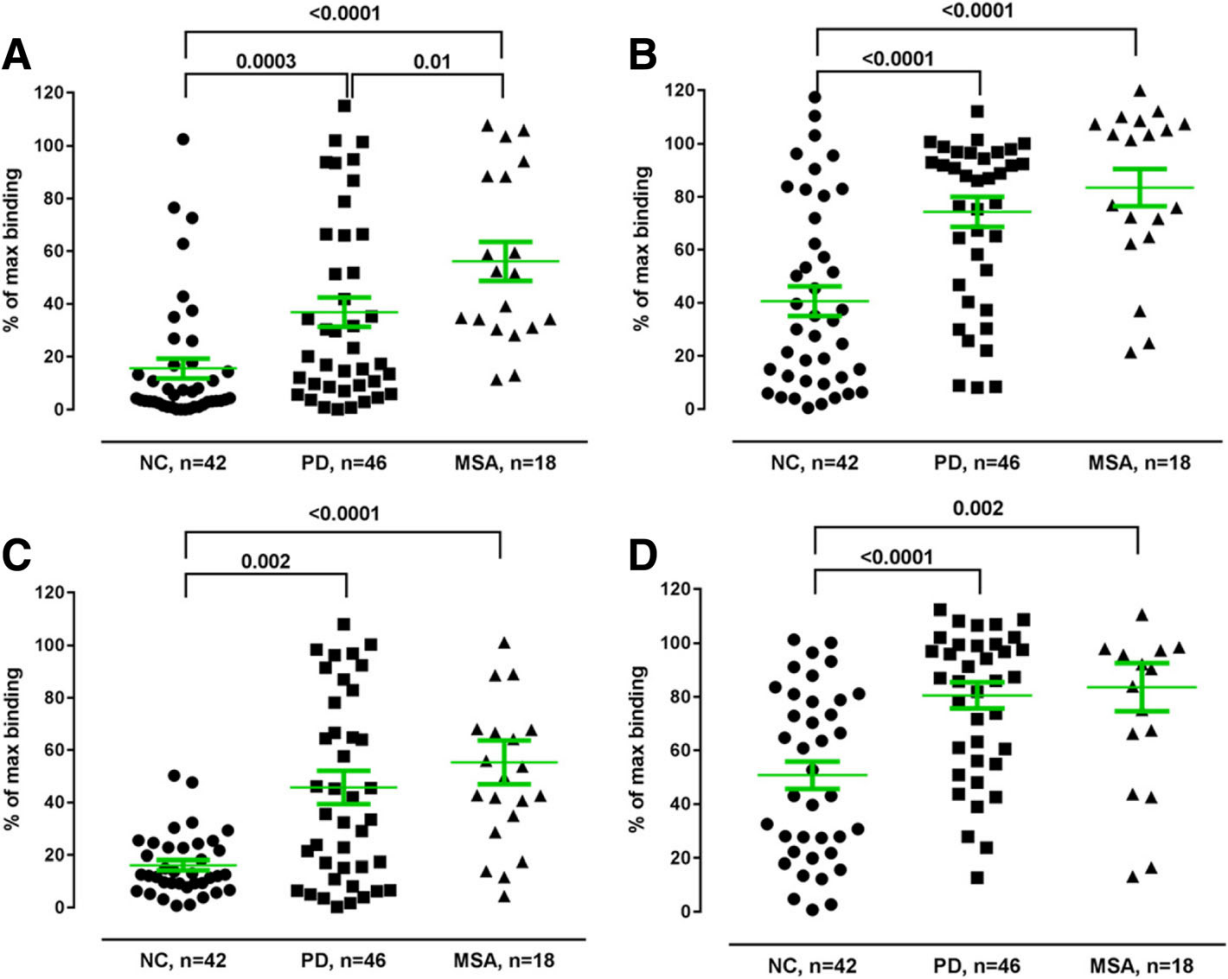
Reduced levels of high affinity anti-phosphorylated (P) α-synuclein NAbs in plasma from MSA and PD patients. Binding of anti-P-α-synuclein NAbs in plasma to immobilized **a**, **b** α-synuclein monomer (10 ng/mL) or **c**, **d** P-α-synuclein (10 ng/mL) in competitive MSD assay in the presence of **a**, **c** 1 nM or **b**, **d** 0.1 nM free P-α-synuclein. Non-specific binding is defined as the binding to the plate in the presence of 100 nM free P-α-synuclein monomer. Maximal binding is defined as the binding observed in the absence of added free α-synuclein monomer. Horizontal bars represent the mean values +/− SEM. Plasma samples from Parkinson’s Disease (PD), Multiple System Atrophy (MSA) patients and normal controls (NC) were tested at 1:400. Significance was tested using Man–Whitney’s U test (*P* < 0.05)

To test binding properties of the anti-P-α-synuclein NAbs, each plasma sample was diluted 1:400 with PBS and preincubated with low 1 nM, 0.1 nM (Fig. 11a–d, respectively) and high concentrations (10 nM) of free P-α-synuclein to saturate the inhibition reaction, followed by incubation on plates coated with 10 ng/ml of immobilized either α-synuclein (11a, b) or P-α-synuclein (11c, d).

The data show that in the presence of 1 nM free P-α-synuclein monomer binding to plates coated with either α-synuclein (Fig. 11a) or P-α-synuclein (Fig. 11c) of approximately 35%–45% and 50–60% of the maximal binding is obtained for anti-P-α-synuclein NAbs in plasma from PD and MSA patients respectively. The corresponding values for normal controls are significantly lower, 15–20%, indicating that these plasma samples contain a significant larger proportion of high affinity anti-P α-synuclein NAbs compared to MSA patients. In the same experiment performed in the presence of 0.1 nM free P-α-synuclein a binding of approximately 80–90% and 70–80% of the maximal binding is obtained for anti-P-α-synuclein NAbs in plasma from PD and MSA patients, respectively. The corresponding values for normal controls are 40–50%% on plates coated with either α-synuclein (Fig. 11b) or P-α-synuclein (Fig. 11d). Under these conditions both MSA and PD are significantly different from healthy controls, meaning that plasma samples from MSA and PD patients contain a significantly lower amount of high affinity anti-P-α-synuclein NAbs compared to normal controls.

## Discussion

Previously, it has been hypothesized that the anti-α-synuclein NAbs levels may be a factor contributing to the pathogenesis of PD [23–29]. However, there was no consistent finding for a change in the anti-α-synuclein NAbs concentration in plasma from PD patients relative to healthy individuals. No study has investigated the avidity of the anti-α-synuclein NAbs in PD and MSA patients, which is possibly more relevant than mere concentrations.

This is the first report on anti-α-synuclein NAbs in MSA, and the first report to link the affinity/avidity of naturally occurring anti-α-synuclein autoantibodies to the clinical phenotypes of PD and MSA. Using a conventional immunoassay set-up, we assessed the NAbs apparent affinity/avidity by fitting one- and two site models to the inhibition curves obtained by adding increasing amounts of α-synuclein monomer to diluted plasma samples. We report that a two-site model proved superior, indicating approximately equal low and high binding components in healthy controls, but a lower fraction of high affinity anti-α-synuclein NAbs in PD patients, and a near absence of the high affinity component in plasma from MSA patients. As with the present results, the antibody affinity and avidity distribution did not vary with age, substantiating our claim that age is not a factor in the present α-synuclein NAb findings. Moreover, we did not find a correlation between disease duration or disease severity and the NAb affinity distribution in PD or MSA nor any differences between males and females (data not shown). This may suggest that the NAbs are not a predictor of disease progression in these diseases, but it does not rule in or out the contribution of NAbs to the control of PD and MSA progression and maybe also as a prognostic tool.

Serine 129 phosphorylated α-synuclein is a major component of neuronal Lewy bodies in PD and of glial cytoplasmatic inclusions in MSA [34]. Phosphorylated α-synuclein can be detected in blood plasma and shows more promise as a diagnostic marker than the nonphosphorylated protein as the levels of P-α-synuclein are higher in PD than in controls [36–38]. We showed near absence of the high affinity anti-P-α-synuclein component in plasma from PD and MSA patients.

The progressive pathological changes in PD and MSA probably start years before the clinical onset of motorsymptoms. The discovery of α-synuclein aggregates in nerve endings of the heart [39], digestive tract (reviewed by Ruffman and Parkkinen [40]), and skin [41, 42] has lent support to the concept of PD as a systemic disease. Braak and coworkers hypothesized that Lewy Body pathology primes in the enteric nervous system and spreads to the brain, suggesting an active retrograde transport of α-synuclein via vagal nerve [43]. Moreover, P-α-synuclein has been detected in the gastrointestinal tract of PD patients and healthy controls [44–47] suggesting that that α-synuclein phosphorylation is a physiologically occurring process. These observations suggest that the immune system is exposed to misfolded, phosphorylated and aggregated forms of α-synuclein already in the early stage in the course of the disease and thus impaired antibody-mediated clearance mechanisms may lead to the progression of the pathology in synucleinopathies. T- and B-lymphocyte subsets are declined in a PD patient cohort during a 5 and 10 year disease course [48] probably contributing to the changes in variability of anti-α-synuclein antibody responses.

NAbs are crucial for microglial phagocytosis of antibody-antigen complexes [49], dissociating aggregates and inhibiting pathological protein aggregation [50] by stabilizing a specific type of an aggregate [51] or by binding an epitope only accessible in a specific protein conformation [52, 53]. Recent observations by Breydo et al. have shown that antibodies can interfere with protein aggregation at substoichiometric concentrations [54]. Interestingly, Inhibition was especially effective in the absence of seeds indicating that early stages in the aggregation pathway were the major targets of the antibody binding.

At this point we may only speculate that high affine α-synuclein antibodies can neutralize the neurotoxic aggregates without interfering with beneficial functions of monomeric α-synuclein. In fact, single chain antibody fragments (scFvs) were isolated from a phage displayed antibody library against the target antigen morphology. These scFvs were proved to bind only to an oligomeric form of α-synuclein and inhibit both aggregation and toxicity of α-synuclein in vitro [55]. Recently, it has also been shown that the robust uptake of α-synuclein oligomer/protofibril selective antibodies by human Central Nervous System (CNS)-derived cells is enhanced by extracellular α-synuclein and mediated via Fcγ receptors [56]. Other monoclonal antibodies have been suggested to promote phagocytosis and lysosomal degradation of α-synuclein [57].

Our finding of decreased plasma α-synuclein concentrations both in PD and MSA groups stands in contrast to previous studies based on traditional sandwich ELISA, which reported increased plasma α-synuclein in these patient groups [36, 58]. The discrepancy may reflect several factors such as sample characteristics, biological sample quality, and especially the nature of the detection antibodies. For example, the different primary antibodies may detect to a varying degree truncated α-synuclein, as well as full-length protein or oligomers forms. Consistent with our results, studies using Western blotting reported decreased plasma α-synuclein levels in PD patients [36, 59]. We speculate that decreased plasma levels of α-synuclein in MSA and PD may reflect the increased α-synuclein load in the brain of this normally cytoplasmic protein [60]. The finding of decreased plasma α-synuclein is further supported by the reduced levels of anti-α-synuclein/NAbs complexes in plasma from PD patients, which was even more pronounced in the MSA group. We can only speculate that the oligomers are transiently present also in healthy individuals, but are adequately cleared by a natural autoimmune mechanism, which is facilitated by the affine binding of NAbs to α-synuclein. PD and MSA occurs mainly in elderly and aging greatly increases the risk of a slow but progressive protein aggregation, thus it can be one of the factors in individuals at risk, which puts a greater stress on the clearance system [28].

The synuclein family consists of three distinct proteins, α-synuclein, β-synuclein, and γ-synuclein sharing many common epitopes [61]. All synuclein protein sequences consist of a highly conserved amino-terminal domain that includes a variable number of 11-residue repeats and a less-conserved carboxy-terminal domain that includes a preponderance of acidic residues. The only significant variations within the repeat domain are the deletion of 11 amino acids (residues 53–63) in β-synuclein. γ-synuclein is smaller than α- and β-synucleins due to a shorter C-terminal region, yet it contains much of the non-Abeta component (NAC) that is missing in β-synuclein [62, 63]. The amino-terminal half of all synucleins is taken up by a highly conserved alpha-helical lipid-binding motif. β-synuclein contains five of these domains, whereas α- and γ-synucleins have six. Despite similar repeat sequences, β-synuclein and γ-synuclein show poor assembly into filaments [61, 64] and have no pathologically disease relation in PD and MSA. In fact, in vitro studies have also shown that both β- and γ-synuclein are able to inhibit fibrillation of α-synuclein [65, 66]. The C-terminal regions of synucleins, although all highly acidic, are rather different. It is probably this structural diversity that leads to differences in NAbs reactivity towards synucleins. Our observations may suggest, the high affinity anti-α-synuclein plasma component, seen in healthy individuals, is directed mainly against C-terminal epitopes or binds to an oligomeric form of α-synuclein. Here, by applying inhibitory ELISA, which detects proteins in a solution, anti-α-synuclein NAbs were shown to be truly selective for physiologically relevant epitopes that are accessible for the antibodies. This may reflect the situation in vivo in healthy individuals, where the high affinity component of anti-α, −β and -γ-synuclein plasma NAbs reacts with mutual epitopes present on α-synuclein.

## Conclusions

The relatively mild, but significant, decrease in high affinity anti-α-synuclein NAbs in plasma of PD patients may parallel the slow progression of PD pathology, whereas the rapidly progressing pathology characteristic of MSA could be attributed to the near absence of high affinity anti-α-synuclein NAbs. This implies that impaired capacity for immune clearance and/or blocking of toxic α-synuclein species may be a factor in these diseases, and support the rationale for immunotherapy with high affine α-synuclein antibodies as a new disease altering approach to treatment [67]. The disease-associated anti α-synuclein NAb alterations in individual autoantibody profiles may also present a new approach for early diagnosis of these neurodegenerative diseases. To what extent the same anti α-synuclein NAb profile is present in cerebrospinal fluid (CSF) and by what mechanisms these autoantibodies seems to play a role in slowing disease development remains to be elucidated.

## Abbreviations

BSA: Bovine Serum Albumin
CNS: Central Nervous System
CSF: Cerebrospinal Fluid
ELISA: Enzyme-linked Immunosorbent Assay
MSA: Multiple System Atrophy
MSD: Meso Scale Discovery
NAbs: Naturally Occurring Autoantibodies
PD: Parkinson’s disease
RT: Room temperature
scFvs: Single Chain Antibody Fragments
TMB: Tetramethylbenzidine

## References

[1] Bhidayasiri R, Ling H (2008). Multiple system atrophy. Neurologist.

[2] Cykowski MD, Coon EA, Powell SZ, Jenkins SM, Benarroch EE, Low PA (2015). Expanding the spectrum of neuronal pathology in multiple system atrophy. Brain.

[3] Stefanova N, Bucke P, Duerr S, Wenning GK (2009). Multiple system atrophy: an update. Lancet Neurol.

[4] Salvesen L, Ullerup BH, Sunay FB, Brudek T, Lokkegaard A, Agander TK (2015). Changes in total cell numbers of the basal ganglia in patients with multiple system atrophy - A stereological study. Neurobiol Dis.

[5] L. Salvesen, K. Winge, T. Brudek, T.K. Agander, A. Lokkegaard, B. Pakkenberg. Neocortical Neuronal Loss in Patients with Multiple System Atrophy: A Stereological Study. Cereb Cortex. 2015. 10.1093/cercor/bhv228 26464477

[6] Joelving FC, Billeskov R, Christensen JR, West M, Pakkenberg B (2006). Hippocampal neuron and glial cell numbers in Parkinson’s disease--a stereological study. Hippocampus.

[7] Dickson DW, Parkinson Disease and Parkinsonism: Neuropathology. Cold Spring Harb Perspect Med. 2012;2. 10.1101/cshperspect.a009258 PMC3405828 22908195

[8] Low PA, Reich SG, Jankovic J, Shults CW, Stern MB, Novak P (2015). Natural history of multiple system atrophy in the USA: a prospective cohort study. Lancet Neurol.

[9] Poewe W, Krismer F (2015). Neurodegenerative disease: Multiple system atrophy - new insight from prospective studies. Nat Rev Neurol.

[10] McCann H, Stevens CH, Cartwright H, Halliday GM (2014). Alpha-Synucleinopathy phenotypes. Parkinsonism Relat Disord.

[11] Kubo M, Kamiya Y, Nagashima R, Maekawa T, Eshima K, Azuma S (2010). LRRK2 is expressed in B-2 but not in B-1 B cells, and downregulated by cellular activation. J Neuroimmunol.

[12] Holmans P, Moskvina V, Jones L, Sharma M, Vedernikov A, The International Parkinson's Disease Genomics Consortium (2013). A pathway-based analysis provides additional support for an immune-related genetic susceptibility to Parkinson’s disease. Hum Mol Genet.

[13] Lutz HU (2012). Naturally occurring antibodies (NAbs).

[14] Avrameas S, Ternynck T, Tsonis IA, Lymberi P (2007). Naturally occurring B-cell autoreactivity: A critical overview. J Autoimmun.

[15] Nagele EP, Han M, Acharya NK, DeMarshall C, Kosciuk MC, Nagele RG (2013). Natural IgG Autoantibodies Are Abundant and Ubiquitous in Human Sera, and Their Number Is Influenced By Age, Gender, and Disease. PLoS One.

[16] Gold M, Pul R, Bach JP, Stangel M, Dodel R (2012). Pathogenic and physiological autoantibodies in the central nervous system. Immunol Rev.

[17] Vargas ME, Watanabe J, Singh SJ, Robinson WH, Barres BA (2010). Endogenous antibodies promote rapid myelin clearance and effective axon regeneration after nerve injury. Proc Natl Acad Sci.

[18] Masliah E, Rockenstein E, Adame A, Alford M, Crews L, Hashimoto M (2005). Effects of Alpha-Synuclein Immunization in a Mouse Model of Parkinson’s Disease. Neuron.

[19] Tran H-T, Chung C-HY, Iba M, Zhang B, Trojanowski J-Q, Luk K-C (2014). Alpha-Synuclein Immunotherapy Blocks Uptake and Templated Propagation of Misfolded Alpha-Synuclein and Neurodegeneration. Cell Rep.

[20] Mandler M, Valera E, Rockenstein E, Weninger H, Patrick C, Adame A (2014). Next-generation active immunization approach for synucleinopathies: implications for Parkinson’s disease clinical trials. Acta Neuropathol.

[21] Mandler M, Valera E, Rockenstein E, Mante M, Weninger H, Patrick C (2015). Active immunization against alpha-synuclein ameliorates the degenerative pathology and prevents demyelination in a model of multiple system atrophy. Mol Neurodegener.

[22] A.L. Bergstrom, P. Kallunki, K. Fog. Development of Passive Immunotherapies for Synucleinopathies. Mov. Disord. 2015;n/a. 10.1002/mds.26481 26704735

[23] Papachroni KK, Ninkina N, Papapanagiotou A, Hadjigeorgiou GM, Xiromerisiou G, Papadimitriou A (2007). Autoantibodies to alpha-synuclein in inherited Parkinsons disease. J Neurochem.

[24] Smith LM, Schiess MC, Coffey MP, Klaver AC, Loeffler DA (2012). Alpha-Synuclein and Anti-Alpha-Synuclein Antibodies in Parkinsons Disease, Atypical Parkinson Syndromes, REM Sleep Behavior Disorder, and Healthy Controls. PLoS One.

[25] Woulfe JM, Duke R, Middeldorp JM, Stevens S, Vervoort M, Hashimoto M (2002). Absence of elevated anti-alpha-synuclein and anti-EBV latent membrane protein antibodies in PD. Neurology.

[26] Gruden MA, Yanamandra K, Kucheryanu VG, Bocharova OR, Sherstnev VV, Morozova-Roche LA (2012). Correlation between Protective Immunity to Alpha-Synuclein Aggregates, Oxidative Stress and Inflammation. Neuroimmunomodulation.

[27] Gruden MA, Sewell RDE, Yanamandra K, Davidova TV, Kucheryanu VG, Bocharov EV (2011). Immunoprotection against toxic biomarkers is retained during Parkinson's disease progression. J Neuroimmunol.

[28] Yanamandra K, Gruden MA, Casaite V, Meskys R, Forsgren L, Morozova-Roche LA (2011). Alpha-Synuclein Reactive Antibodies as Diagnostic Biomarkers in Blood Sera of Parkinson’s Disease Patients. PLoS One.

[29] Besong-Agbo D, Wolf E, Jessen F, Oechsner M, Hametner E, Poewe W (2013). Naturally occurring alpha-synuclein autoantibody levels are lower in patients with Parkinson disease. Neurology.

[30] Calne DB, Snow BJ, Lee C (1992). Criteria for diagnosing Parkinson’s disease. Ann Neurol.

[31] Gilman S, Wenning GK, Low PA, Brooks DJ, Mathias CJ, Trojanowski JQ (2008). Second consensus statement on the diagnosis of multiple system atrophy. Neurology.

[32] Bastarache JA, Koyama T, Wickersham NE, Ware LB (2014). Validation of a multiplex electrochemiluminescent immunoassay platform in human and mouse samples. J Immunol Methods.

[33] Dunn-Walters DK, Banerjee M, Mehr R (2003). Effects of age on antibody affinity maturation. Biochem Soc Trans.

[34] Fujiwara H, Hasegawa M, Dohmae N, Kawashima A, Masliah E, Goldberg MS (2002). Alpha-Synuclein is phosphorylated in synucleinopathy lesions. Nat Cell Biol.

[35] Inglis KJ, Chereau D, Brigham EF, Chiou SS, Schöbel S, Frigon NL (2009). Polo-like Kinase 2 (PLK2) Phosphorylates Alpha-Synuclein at Serine 129 in Central Nervous System. J Biol Chem.

[36] Duran R, Barrero FJ, Morales B, Luna JD, Ramirez M, Vives F (2010). Plasma alpha-synuclein in patients with Parkinson’s disease with and without treatment. Mov Disord.

[37] Foulds PG, Mitchell JD, Parker A, Turner R, Green G, Diggle P (2011). Phosphorylated alpha-synuclein can be detected in blood plasma and is potentially a useful biomarker for Parkinson’s disease. FASEB J.

[38] Foulds PG, Diggle P, Mitchell JD, Parker A, Hasegawa M, Masuda-Suzukake M (2013). A longitudinal study on alpha-synuclein in blood plasma as a biomarker for Parkinson’s disease. Sci Rep.

[39] Iwanaga K, Wakabayashi K, Yoshimoto M, Tomita I, Satoh H, Takashima H (1999). Lewy body-type degeneration in cardiac plexus in Parkinson’s and incidental Lewy body diseases. Neurology.

[40] C. Ruffmann,L. Parkkinen. Gut Feelings About Alpha-Synuclein in Gastrointestinal Biopsies: Biomarker in the Making? Mov. Disord. 2016;n/a. 10.1002/mds.26480 PMC4755164 26799450

[41] Rodriguez-Leyva I, Calderon-Garciduenas AL, Jimenez-Capdeville ME, Renteria-Palomo AA, Hernandez-Rodriguez HG, Valdes-Rodriguez R (2014). Alpha-Synuclein inclusions in the skin of Parkinson’s disease and parkinsonism. Ann Clin Transl Neurol.

[42] Zange L, Noack C, Hahn K, Stenzel W, Lipp A (2015). Phosphorylated alpha-synuclein in skin nerve fibres differentiates Parkinson’s disease from multiple system atrophy. Brain.

[43] Braak H, de Vos RAI, Bohl J, Del Tredici K (2006). Gastric alpha-synuclein immunoreactive inclusions in Meissner’s and Auerbach’s plexuses in cases staged for Parkinson's disease-related brain pathology. Neurosci Lett.

[44] Lebouvier T, Chaumette T, Damier P, Coron E, Touchefeu Y, Vrignaud S (2008). Pathological lesions in colonic biopsies during Parkinson’s disease. Gut.

[45] Lebouvier T, Neunlist M, Bruley des Varannes S, Coron E, Drouard A, N’Guyen JM (2010). Colonic Biopsies to Assess the Neuropathology of Parkinson’s Disease and Its Relationship with Symptoms. PLoS One.

[46] Hilton D, Stephens M, Kirk L, Edwards P, Potter R, Zajicek J (2014). Accumulation of alpha-synuclein in the bowel of patients in the pre-clinical phase of Parkinson’s disease. Acta Neuropathol.

[47] Visanji NP, Marras C, Kern DS, Al Dakheel A, Gao A, Liu LW (2015). Colonic mucosal α-synuclein lacks specificity as a biomarker for Parkinson disease. Neurology.

[48] Gruden MA, Sewell RDE, Yanamandra K, Davidova TV, Kucheryanu VG, Bocharov EV (2011). Immunoprotection against toxic biomarkers is retained during Parkinson’s disease progression. J Neuroimmunol.

[49] Bae EJ, Lee HJ, Rockenstein E, Ho DH, Park EB, Yang NY (2012). Antibody-Aided Clearance of Extracellular Alpha-Synuclein Prevents Cell-to-Cell Aggregate Transmission. J Neurosci.

[50] Hard T, Lendel C (2012). Inhibition of Amyloid Formation. J Mol Biol.

[51] Ladiwala AR, Bhattacharya M, Perchiacca JM, Cao P, Raleigh DP, Abedini A (2012). Rational design of potent domain antibody inhibitors of amyloid fibril assembly. Proc Natl Acad Sci.

[52] Legleiter J, Lotz GP, Miller J, Ko J, Ng C, Williams GL (2009). Monoclonal Antibodies Recognize Distinct Conformational Epitopes Formed by Polyglutamine in a Mutant Huntingtin Fragment. J Biol Chem.

[53] Dumoulin M, Last AM, Desmyter A, Decanniere K, Canet D, Larsson G (2003). A camelid antibody fragment inhibits the formation of amyloid fibrils by human lysozyme. Nature.

[54] Breydo L, Morgan D, Uversky VN (2016). Pseudocatalytic Antiaggregation Activity of Antibodies: Immunoglobulins can Influence Alpha-Synuclein Aggregation at Substoichiometric Concentrations. Mol Neurobiol.

[55] Emadi S, Barkhordarian H, Wang MS, Schulz P, Sierks MR (2007). Isolation of a Human Single Chain Antibody Fragment Against Oligomeric Alpha-Synuclein that Inhibits Aggregation and Prevents Alpha-Synuclein-induced Toxicity. J Mol Biol.

[56] Gustafsson G, Eriksson F, Moeller C, Fonseca TL, Outeiro TF, Lannfelt L, et al. Cellular Uptake of Alpha-Synuclein Oligomer-Selective Antibodies is Enhanced by the Extracellular Presence of Alpha-Synuclein and Mediated via FcGamma Receptors. Cell Mol Neurobiol. 2016:1–11. 10.1007/s10571-016-0352-5 PMC11482100 26961542

[57] Masliah E, Rockenstein E, Mante M, Crews L, Spencer B, Adame A (2011). Passive Immunization Reduces Behavioral and Neuropathological Deficits in an Alpha-Synuclein Transgenic Model of Lewy Body Disease. PLoS One.

[58] Lee PH, Lee G, Park HJ, Bang OY, Joo IS, Huh K (2006). The plasma alpha-synuclein levels in patients with Parkinson disease and multiple system atrophy. J Neural Transm.

[59] Li QX, Mok SS, Laughton KM, McLean CA, Cappai R, Masters CL (2007). Plasma alpha-synduclein is decreased in subjects with Parkinson’s disease. Exp Neurol.

[60] Brudek T, Winge K, Bredo Rasmussen N, Bahl Czarna JM, Tanassi J, Klitmoller Agander T (2016). Altered Alpha-Synuclein, Parkin, and Synphilin Isoform Levels in Multiple System Atrophy Brains. J Neurochem.

[61] Lavedan C (1998). The synuclein family. Genome Res.

[62] George J (2001). The synucleins. Genome Biol.

[63] E.H. Norris, B.I. Giasson, and V.M.Y. Lee. Alpha-Synuclein: Normal Function and Role in Neurodegenerative Diseases. In: Current Topics in Developmental Biology, Stem Cells in Development and Disease. P.S. Gerald, editor. Academic Press, 2004, 17–54. 10.1016/S0070-2153(04)60002-0 15094295

[64] Goedert M (2001). Alpha-synuclein and neurodegenerative diseases. Nat Rev Neurosci.

[65] Uversky VN, Li J, Souillac P, Millett IS, Doniach S, Jakes R (2002). Biophysical Properties of the Synucleins and Their Propensities to Fibrillate: INHIBITION OF ALPHA-SYNUCLEIN ASSEMBLY BY BETA- AND GAMMA-SYNUCLEINS. J Biol Chem.

[66] Park JY, Lansbury PT (2003). Beta-Synuclein Inhibits Formation of Alpha-Synuclein Protofibrils: A Possible Therapeutic Strategy against Parkinson’s Disease. Biochemistry.

[67] Athauda D, Foltynie T (2015). The ongoing pursuit of neuroprotective therapies in Parkinson disease. Nat Rev Neurol.

